# Degradation Mechanism of Low-Heat Portland Cement Paste Under Semi-Immersion Magnesium Sulfate Attack with Wetting–Drying Cycles

**DOI:** 10.3390/ma19204373

**Published:** 2026-10-18

**Authors:** Boran Yang, Yu Chen, Chunmeng Jiang, Guo Chen, Alimu Maimaiti

**Affiliations:** 1 College of Hydraulic and Civil Engineering, Xinjiang Agricultural University, Urumqi 830052, China; 19825962116@163.com (B.Y.); 15756361493@163.com (Y.C.); m15883704037@163.com (G.C.); alm31377@outlook.com (A.M.); 2 Xinjiang Key Laboratory of Hydraulic Engineering Security and Water Disasters Prevention, Urumqi 830052, China

**Keywords:** low-heat Portland cement, sulfate-magnesium combined attack, semi-immersion, wetting–drying cycles, M-S-H, durability

## Abstract

Concrete structures in the saline–alkaline regions of western China frequently suffer from combined sulfate and magnesium attack. Semi-immersion and wetting–drying cycles in the water-level fluctuation zone make up a typical service scenario. This study investigates the performance evolution and microstructural degradation of low-heat Portland cement (LHPC) paste exposed to 5% MgSO_4_ solution over 90 days under semi-immersion with wetting–drying cycles, using full immersion as a macroscopic reference. The results show that MgSO_4_ attack under this regime exhibits a two-stage behavior, featuring early strengthening followed by later softening. In the early stage, Mg^2+^ is inferred to react with Ca(OH)_2_ to form a brucite layer that fills pores and impedes ion ingress. Once this layer is compromised, Mg^2+^ is inferred to penetrate the matrix and cause decalcification of the C-S-H gel, converting it into non-cementitious M-S-H. Under semi-immersion with wetting–drying cycles, capillary suction and evaporative concentration are considered to enrich Mg^2+^ in the evaporation zone, accelerating this transformation and leading to a 29.03% loss in compressive strength at 90 days, exceeding the 22.25% loss under full immersion. Microscopic characterizations focusing on the semi-immersed specimens indicate that the loss of cementitiousness associated with M-S-H formation is likely an important contributor to the strength loss, while pore coarsening appears to be a secondary effect. The XRD patterns are consistent with M-S-H formation, and the SEM-EDS results show Mg and Si enrichment and Ca depletion in the evaporation zone, providing complementary evidence. The low C_3_A content of LHPC is expected to limit ettringite-related expansion, whereas the abundant C-S-H gel derived from C_2_S hydration may provide a substantial substrate for Mg^2+^ attack, making structural softening the dominant deterioration risk under the tested conditions. For the specific LHPC paste and exposure conditions investigated, strength degradation is recommended as a more informative indicator than expansion for magnesium sulfate attack. These findings help to clarify the degradation mechanism of LHPC under accelerated semi-immersion conditions. The limitations of the accelerated paste test should be considered when extrapolating these results to field service conditions.

## 1. Introduction

Low-heat Portland cement (LHPC) is a type of cement with dicalcium silicate (C_2_S) as its predominant mineral phase and a tricalcium aluminate (C_3_A) content strictly controlled below 6%. This mineral composition endows LHPC with low heat of hydration, high later-age strength, and excellent volume stability [1]. Leveraging these performance advantages, LHPC has been widely used in major hydraulic projects in China, such as Wudongde, Baihetan, and Xiluodu, encompassing both dam bodies and critical components including spillways and stilling basins. In addition to hydraulic structures, LHPC is also widely employed in mass foundation concrete for large-scale infrastructure, where volume stability and low heat generation are equally critical. With the sustained advancement of China’s western development strategy, an increasing number of hydraulic and transportation infrastructure projects are expected to adopt LHPC as the primary cementitious material. However, the western regions of China, including Xinjiang, Gansu, and Qinghai, are characterized by extensive salt lakes and saline soils, with generally high concentrations of SO_4_^2−^ and Mg^2+^ in groundwater and soil [2,3]. These aggressive ions pose a serious threat to the long-term durability of cement-based materials. Therefore, the service performance of LHPC under the complex aggressive environments in western China requires systematic investigation.

To understand the behavior of LHPC under aggressive environments, it is useful to compare its mineralogy with that of ordinary Portland cement (OPC). OPC is dominated by C_3_S and C_3_A, whereas LHPC is characterized by a predominance of C_2_S and a lower C_3_A content. This difference is directly reflected in the hydration product assemblage, where OPC produces substantial amounts of calcium hydroxide (CH) and rapidly forms ettringite (AFt) in the early stage, while LHPC generates less CH and exhibits a continuous increase in C-S-H gel content with age [4]. When exposed to Na_2_SO_4_ attack, LHPC exhibits excellent sulfate resistance because its extremely low C_3_A content significantly suppresses the expansive formation of ettringite. However, when Na_2_SO_4_ is replaced by MgSO_4_ as the aggressive agent, the reaction mechanism changes fundamentally. Mg^2+^ not only consumes CH to form brucite (Mg(OH)_2_) but, more critically, directly attacks C-S-H gel through a Ca^2+^ exchange reaction, converting it into non-cementitious magnesium silicate hydrate (M-S-H) and consequently leading to loss of cementitiousness and matrix softening [5,6,7]. Over prolonged service, calcium leaching further exacerbates the decalcification of C-S-H, rendering the Mg^2+^ exchange reaction more pronounced [8]. For LHPC, the abundant C-S-H gel derived from hydration of its high C_2_S content serves as the primary source of later-age strength, yet simultaneously provides a substantial substrate for Mg^2+^-induced decalcification, rendering its deterioration risk in magnesium-rich environments markedly higher than in sodium-rich environments. Jiang et al. [9] reported that the compressive strength loss of LHPC after 360 days of exposure to MgSO_4_ solution was 3.1 times that in Na_2_SO_4_ solution. Thus, the mineral composition of LHPC exhibits a double-edged sword effect in magnesium-rich environments, where low C_3_A suppresses expansion-induced damage while high C_2_S amplifies the risk of structural softening.

Most existing studies on sulfate attack on cement-based materials have been conducted under full immersion conditions [10]. In practice, however, hydraulic concrete structures are often partially immersed, with a portion submerged in the aggressive solution and the remainder exposed to the atmosphere. Under semi-immersion, capillary suction draws the solution upward from the immersion zone, and continuous evaporation leads to significant salt enrichment near the wetting–drying interface [11,12,13]. Concurrently, the cyclic saturation-desaturation stresses induce microstructural fatigue damage. Chen et al. [14] reported that pore structure damage in cement paste evolves in a multi-scale manner under alternating freeze–thaw and sulfate attack. The wetting–drying process under semi-immersion exerts a similar cyclic fatigue effect on the pore structure. Studies have shown that under semi-immersion in MgSO_4_ solution, the evaporation zone primarily undergoes physical attack by sulfate crystallization, while the immersion zone is dominated by chemical attack [15,16]. Lowering the relative humidity promotes the formation of narrow supersaturated evaporation zones, accelerating salt crystallization and surface spalling. This process is significantly influenced by ambient temperature [17,18]. The deterioration rate under wetting–drying cycles is markedly higher than that under full immersion [19]. Moreover, the ion transport mechanism is no longer steady-state diffusion driven by concentration gradients, but rather non-steady-state capillary suction and evaporative concentration. Under full immersion, SO_4_^2−^ and Mg^2+^ enter the matrix through steady-state diffusion driven by concentration gradients. Under semi-immersion, however, non-steady-state capillary suction is the dominant driving force. It draws solution upward through capillary pores in the unsubmerged region, while water evaporates at the exposed surface and causes aggressive ions to concentrate significantly at the evaporation front [20,21]. In addition, the cation type in the external environment and supplementary cementitious materials greatly influence the stability of erosion products and the chemical environment of the pore solution [22,23].

Semi-immersion and wetting–drying cycles have been widely used in sulfate attack studies [24,25,26,27]. However, most of these studies focused on OPC or blended cement systems, and the spatial evolution of degradation in LHPC under capillary-dominated transport remains unclear. Although some studies have begun to address sulfate attack under semi-immersion conditions, systematic investigation of LHPC under combined sulfate and magnesium attack with semi-immersion and wetting–drying cycles, which is a representative service condition in western China, is still scarce. Specifically, three critical issues remain unclear. First, almost all existing studies on magnesium sulfate attack have been conducted under full immersion, and it is unknown whether the non-steady-state ion transport driven by capillary suction under semi-immersion leads to different distributions of attack products and damage modes. Second, although the expansion rate of cement-based materials under magnesium sulfate attack is known to be much lower than that under sodium sulfate attack [8], the relationship between strength degradation and expansion response in LHPC, an inherently low-expansion cement, has yet to be clarified. Third, the high C_2_S content of LHPC, while beneficial to later-age strength development, provides more C-S-H gel as a substrate for Mg^2+^ attack. Whether this hydration product characteristic becomes a structural vulnerability under the accelerated Mg^2+^ supply driven by wetting–drying cycles remains to be determined. The quantitative difference in deterioration rate compared with full immersion conditions also requires further investigation.

Therefore, this study systematically investigates the deterioration behavior of LHPC paste exposed to 5% MgSO_4_ solution over 90 days under semi-immersion coupled with wetting–drying cycles. Full immersion is included as a macroscopic baseline for comparison of deterioration rates, while microscopic characterizations, including X-ray diffraction (XRD), scanning electron microscopy (SEM), and mercury intrusion porosimetry (MIP), are concentrated on the semi-immersed specimens to reveal the unique degradation features induced by capillary suction and evaporative concentration. The aim is to elucidate the deterioration mechanism of LHPC under this representative service condition and to provide an experimental basis for the durability design of low-heat cement-based materials in aggressive salt-rich environments. In contrast to the previous studies on full immersion [2,9], the present work focuses on the semi-immersion regime and separates the degradation spatially into the immersion and evaporation zones.

## 2. Materials and Methods

### 2.1. Materials and Specimen Fabrication

The cement used in this study was low-heat Portland cement (LHPC. Jiahua Special Cement Co., Ltd., Leshan, China), with its chemical and mineral composition given in Table 1 and its physical and mechanical properties in Table 2. The C_3_A content of this cement is 2.1% and the C_2_S content is 43.9%, which are consistent with the typical mineralogical characteristics of low-heat cement. The aggressive solution was prepared from anhydrous MgSO_4_ (Tianjin Zhiyuan Chemical Reagent Co., Ltd., Tianjin, China) at a concentration of 5%.

Cement paste was prepared with a water-to-binder ratio of 0.40. The paste was cast into Φ50 mm × 100 mm cylindrical molds and 25 mm × 25 mm × 280 mm prismatic molds. For the prismatic molds, copper measuring studs were embedded at the two ends for linear expansion measurement. After casting, the specimens were cured under standard conditions (20 °C, 98% RH) for 24 h and then demolded. They were subsequently transferred to saturated lime water at 20 °C and cured until 90 days of age, after which they were immersed in the MgSO_4_ solution.

### 2.2. Experimental Design

Two exposure conditions were designed, as shown in Table 3. The first condition was full immersion, designated as Group F, in which the specimens were continuously and fully immersed in 5% MgSO_4_ solution throughout the test period. The second condition was semi-immersion coupled with wetting–drying cycles, designated as Group S, in which the specimens were immersed to half of their height. The full immersion condition was intended to simulate the steady-state aggressive environment of concrete in the submerged zone, while the semi-immersion with wetting–drying cycles was intended to simulate the aggressive environment of concrete in the water-level fluctuation zone, where capillary suction and evaporative concentration act together. To facilitate subsequent microscopic characterization and mechanistic analysis, the semi-immersed specimens were divided along the height direction into two typical zones. The portion always below the liquid level was designated as the immersion zone, and the portion exposed to the atmosphere, where capillary suction drew up the solution and salt accumulated, was designated as the evaporation zone. Subsequent SEM, XRD, and MIP test samples were taken from these two zones separately. Given that the microstructural response of LHPC under full MgSO_4_ immersion has been extensively addressed in previous studies [2,9], the microscopic characterizations in this work are primarily dedicated to the semi-immersed specimens, particularly the evaporation zone, to reveal the unique deterioration features induced by the wetting–drying regime.

**Figure 1:**
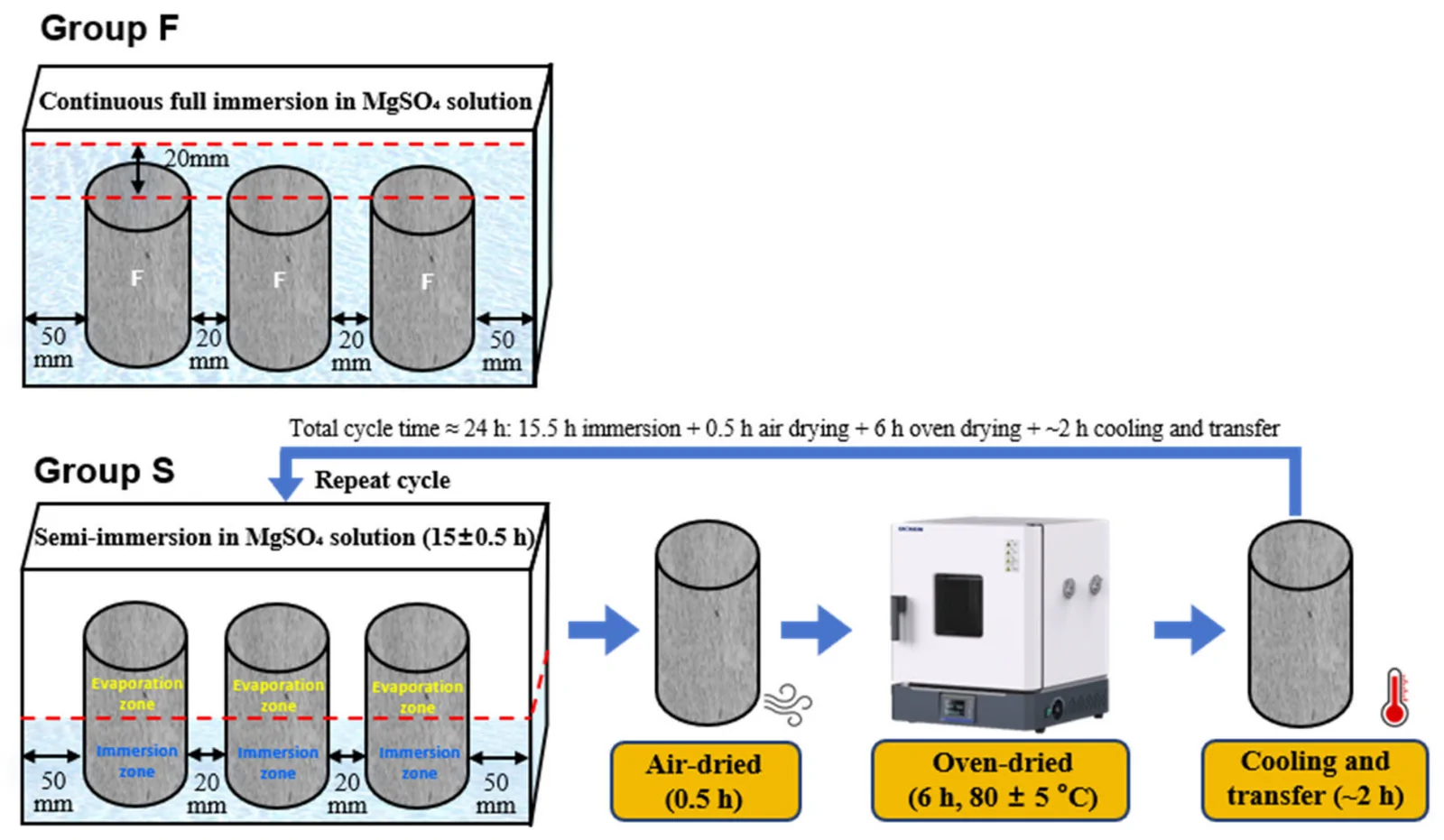
Schematic of the test procedure for the two exposure groups.

For Group S, each cycle lasted approximately 24 h, consisting of 15.5 h of immersion, 0.5 h of natural air drying, 6 h of oven drying at 80 ± 5 °C in a forced-air drying oven (DHG-9045B, LICHEN, Shanghai, China), and approximately 2 h for cooling and transfer. The total effective treatment time was 22 h. For both groups, specimens were sampled every 30 days for various performance tests, and the total test duration was 90 days. For the semi-immersed specimens, sampling was carried out immediately after the immersion stage of the wetting–drying cycle, that is, before the subsequent air drying and oven drying stages. This ensured that the specimens were in a saturated state when sampled, consistent with the state of the fully immersed specimens at the time of sampling. The aggressive solution for both groups was 5% MgSO_4_ solution, which was renewed weekly to maintain relatively stable concentrations of aggressive ions. The test procedure is illustrated in Figure 1. This accelerated regime was adapted from GB/T 50082-2024 [28] and is intended to amplify the differences between exposure conditions within a short test period. It should be regarded as an accelerated mechanistic study rather than a direct simulation of field service conditions. Also, the wetting–drying cycles involve both chemical attack and thermal drying, and the absence of a control group without MgSO_4_ means that these effects cannot be fully separated in this study. The relationship between this regime and realistic field exposure is discussed in Section 4.

### 2.3. Characterization Methods

All macroscopic measurements, including mass loss, porosity, compressive strength, and linear expansion, were performed on three parallel specimens. The results are presented as the mean ± standard deviation (*n* = 3). Error bars in the subsequent figures represent the standard deviation. For microstructural characterization, samples were taken from the surface layer of the immersion zone and the evaporation zone of the semi-immersed specimens, respectively.

#### 2.3.1. Mass Loss Measurement

The mass loss of specimens was monitored throughout the exposure period using three fixed parallel specimens for each group. At each exposure age, the specimens were removed from the solution, surface dried with a damp cloth to a saturated surface-dry condition, and then weighed. The mass loss rate *W_t_* was calculated by Equation (1).

(1) $$W_{t}=\frac{m_{0}-m_{t}}{m_{0}}\times 100\%$$

where *m_t_* and *m*_0_ are the masses of the specimen after and before exposure, respectively, measured in the saturated surface-dry condition (g).

#### 2.3.2. Open Porosity Measurement

Three parallel cylindrical specimens were used for open porosity measurement. The open porosity was determined by the water saturation method. The specimens were oven-dried at 105 °C to constant mass and weighed to obtain the dry mass *m_d_*. They were then placed in a vacuum saturator under an absolute pressure of 65 kPa for 60 min to achieve full saturation. After saturation, the specimens were removed, surface-dried with a damp cloth, and weighed to obtain the saturated surface-dry mass *m_s_*. The specimen volume *V* was measured by the drainage method. The open porosity *P_o_* was calculated from the water absorption according to Equation (2), and the average value of the three parallel specimens was taken as the test result.

(2) $$P_{o}=\frac{m_{s}-m_{d}}{V}\times 100\%$$

#### 2.3.3. Compressive Strength Test

The cylindrical specimens were tested under uniaxial compression using an electronic universal testing machine at a loading rate of 0.10 mm/min. The failure load was recorded and the compressive strength was calculated. The average value of three parallel specimens was taken as the compressive strength at each exposure age. The compressive strength loss rate was then calculated by Equation (3):

(3) $$\Delta \sigma t=\frac{f_{0}-f_{t}}{f_{0}}\times 100$$

where Δσt is the compressive strength loss rate after *t* days of exposure (%), and *f*_0_ and *f_t_* are the compressive strengths of the specimens before and after *t* days of exposure, respectively (MPa). Compressive strength was measured on the whole cylindrical specimen.

#### 2.3.4. Linear Expansion Measurement

Three parallel specimens were used for linear expansion measurement. The length of the specimens was measured using a length comparator with a digital dial gauge accurate to 0.001 mm, following the method specified in JC/T 603-2004 [29]. The linear expansion rate was calculated by Equation (4), and the average value of the three specimens was taken as the test result.

(4) $$\Delta l_{t}=\frac{l_{t}-l_{0}}{l_{0}}\times 100$$

where *l*_0_ and *l_t_* are the lengths of the specimen before and after *t* days of exposure, respectively (mm).

#### 2.3.5. SEM-EDS Analysis

Specimens for microstructural observation were taken from the surface layer of the eroded cement paste, with dimensions of approximately 0.5 cm^3^. After gold sputter coating, the samples were examined using a Hitachi SU8010 scanning electron microscope (Hitachi, Tokyo, Japan) at an accelerating voltage of 15 kV. The elemental composition of selected regions was determined using an energy-dispersive X-ray spectroscopy (EDS) detector coupled to the SEM (Hitachi, Tokyo, Japan). EDS spectra were collected by point/area scanning from the typical reaction product regions in the immersion and evaporation zones.

#### 2.3.6. Phase Composition Analysis

Powder samples for XRD analysis were collected from the surface layer of the specimens. Phase identification was performed using a D8 X-ray diffractometer (Bruker, Berlin, Germany) with Cu Kα radiation (40 kV, 40 mA), at a scanning speed of 10°/min, a step size of 0.02°, and a 2θ range of 10° to 50°.

#### 2.3.7. Pore Structure Analysis

Samples for pore structure analysis were taken from the surface layer of the specimens, with dimensions of approximately 0.5 cm^3^. The samples were dried in the same forced-air drying oven (DHG-9045B, LICHEN, Shanghai, China) at 60 °C to constant mass and then subjected to pore structure analysis using an AutoPore IV 9510 automatic mercury intrusion porosimeter (Micromeritics, Norcross, GA, USA). The contact angle between mercury and the sample was set at 130°, and the surface tension was 0.485 N/m.

## 3. Results

### 3.1. Mass Loss

**Figure 2:**
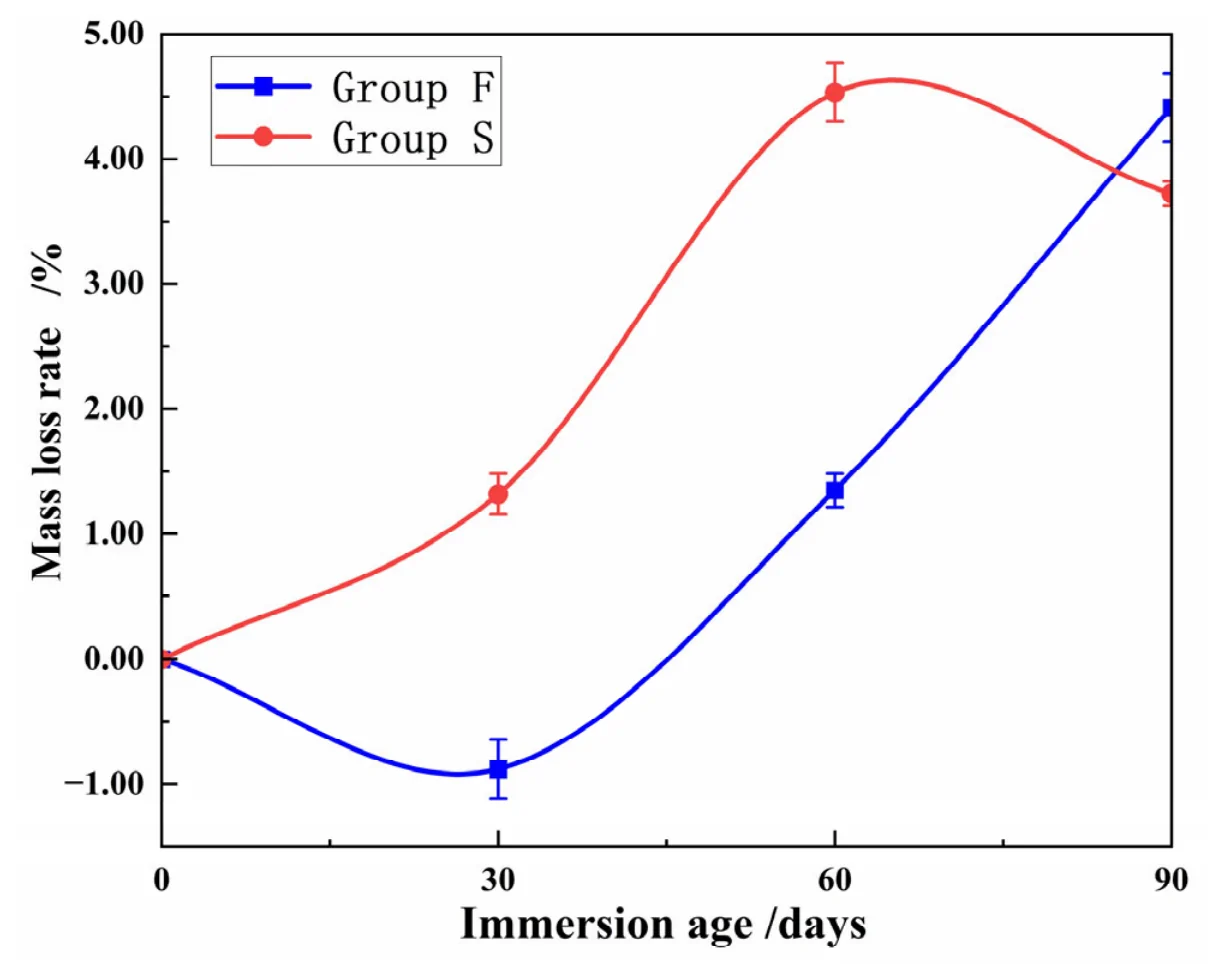
Mass loss rate of LHPC paste under different exposure conditions.

Figure 2 presents the mass loss rate of LHPC paste specimens exposed to 5% MgSO_4_ solution under full immersion and semi-immersion with wetting–drying cycles. Under full immersion, the mass loss rate was −0.88% at 30 days, indicating a slight mass gain. It became positive at 60 days and further increased to 4.41% at 90 days. The transition from negative to positive occurred between 30 and 60 days. The early-stage mass gain indicates that the rate of erosion product deposition within pores exceeded the rate of surface material spalling, leading to a net increase in specimen mass. With prolonged exposure, surface spalling gradually became dominant, resulting in a continuous increase in mass loss rate. Under semi-immersion with wetting–drying cycles, the mass loss rate was 1.32% at 30 days, rose to 4.53% at 60 days, and then decreased to 3.72% at 90 days. The mass loss of Group S was higher than that of Group F at 30 and 60 days, but lower at 90 days. The decrease from 60 to 90 days should be interpreted with caution, as the measured mass change represents the net result of competing processes, including surface spalling, deposition of reaction products, and salt crystallization.

### 3.2. Open Porosity

**Figure 3:**
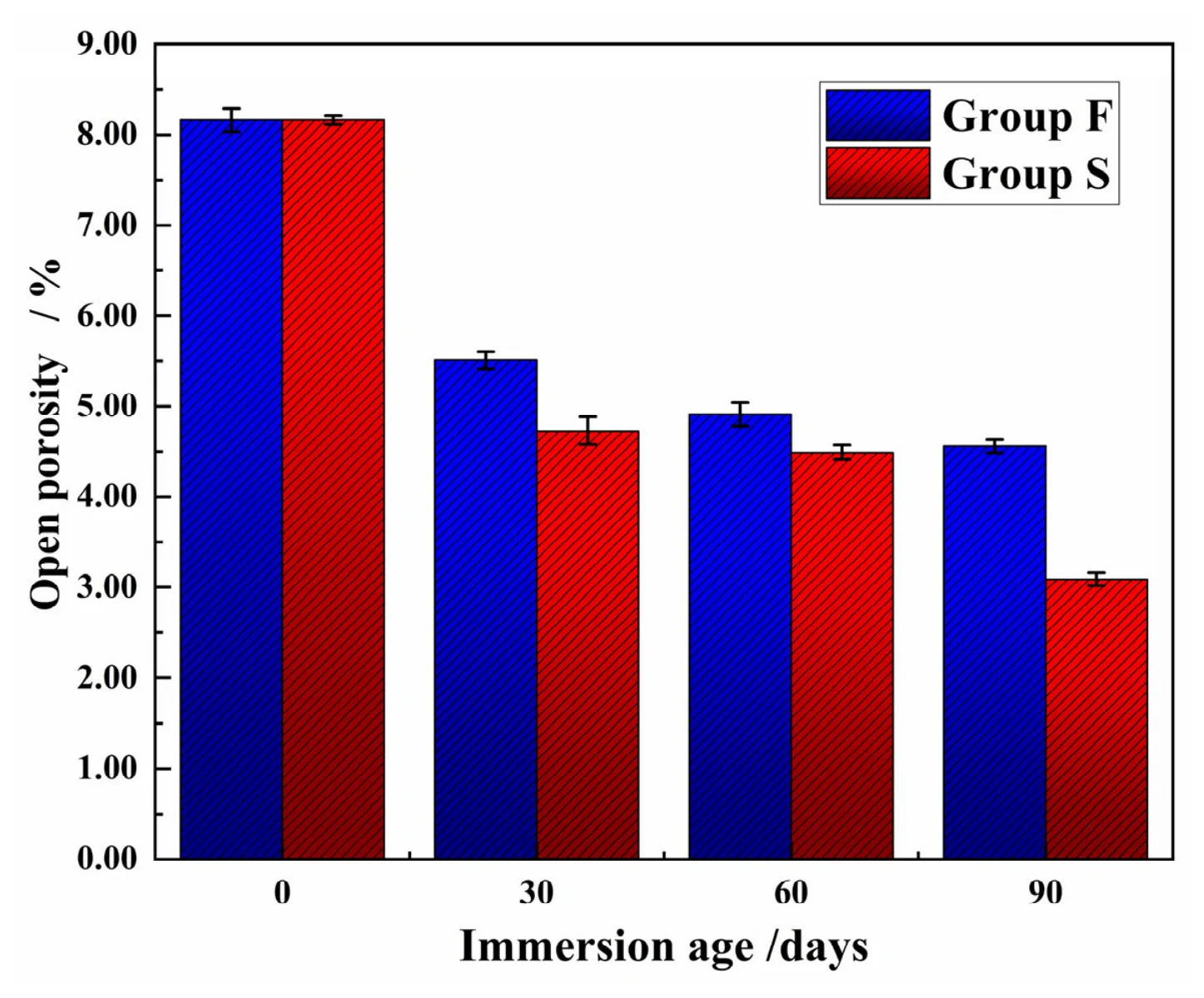
Open porosity of LHPC paste under different exposure conditions.

The evolution of open porosity is illustrated in Figure 3. Under full immersion, the open porosity decreased rapidly at the early stage, with a total reduction of 2.65 percentage points, and then the decrease gradually slowed down, reaching 4.56% at 90 days. Under semi-immersion with wetting–drying cycles, the open porosity dropped to 4.73% at 30 days, lower than that of the full-immersion specimens at the same age. Unlike the full-immersion group, in which the open porosity reduction tended to stabilize after the early stage, the semi-immersion group exhibited a further marked decrease between 60 and 90 days, reaching a final value of 3.09%, approximately two-thirds of that of the full-immersion group at the same age. In comparison, the semi-immersion group consistently showed lower open porosity than the full-immersion group at all exposure ages, with differences of 0.78 percentage points at 30 days and 1.47 percentage points at 90 days.

The continuous decrease in open porosity under both conditions indicates that attack products continued to deposit within the pores. Under full immersion, the open porosity reduction was mainly concentrated in the early stage, with the later stage approaching saturation. Under semi-immersion, however, the pore-filling effect persisted into the later stage due to the continuous supply of aggressive ions driven by wetting–drying cycles, leading to a further reduction in open porosity. It is worth noting that the simultaneous decrease in open porosity and increase in mass loss rate are not contradictory [30,31]. Open porosity reflects changes in the open pore volume of the whole specimen, where pore filling by degradation products dominates the trend. In contrast, mass loss includes the contribution of surface spalling, and when the spalling rate exceeds the mass gain from internal product deposition, the mass loss rate turns positive.

### 3.3. Compressive Strength

**Figure 4:**
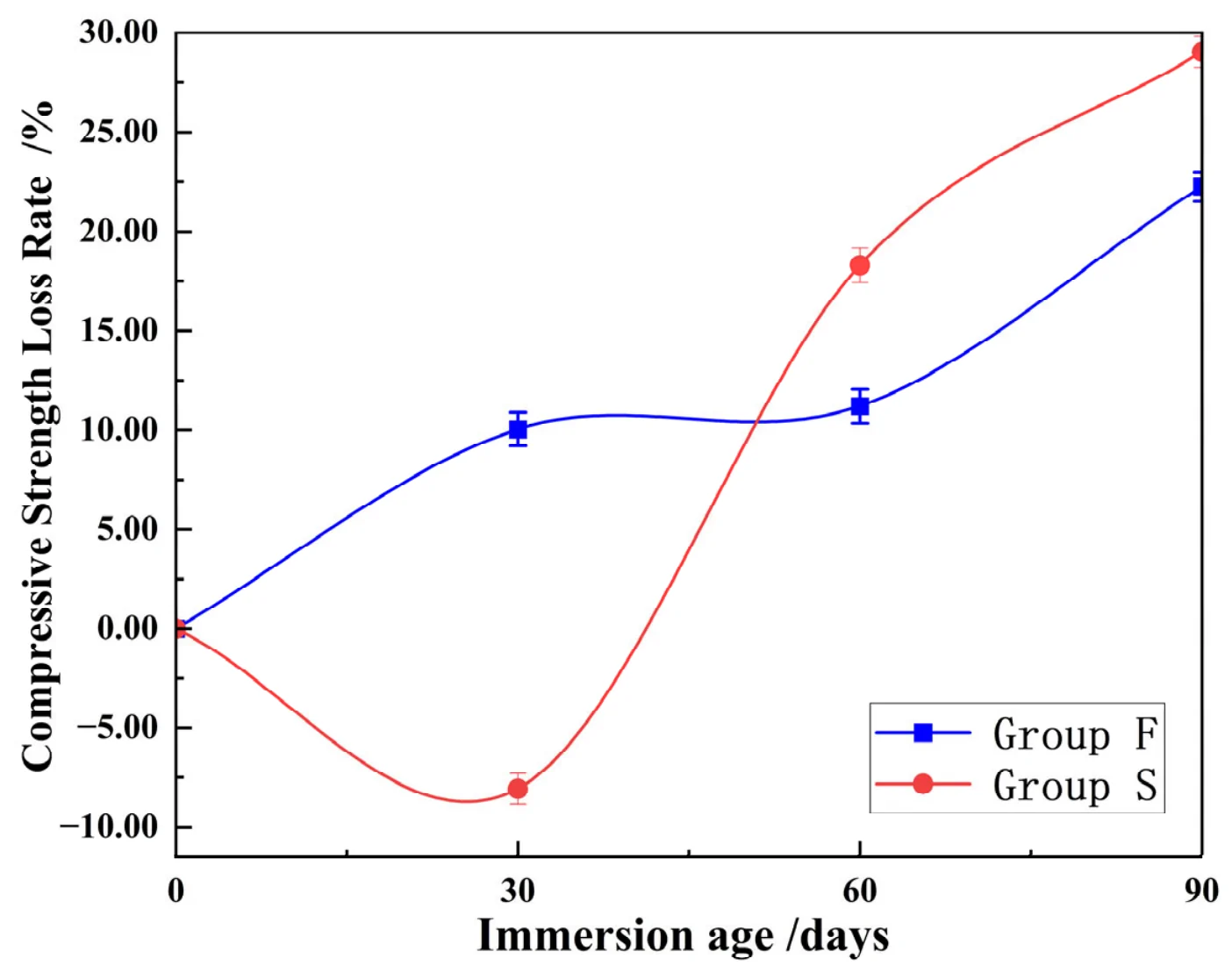
Compressive strength loss rate of LHPC paste under different exposure conditions.

Figure 4 presents the compressive strength loss rate of LHPC paste specimens under the two exposure conditions.

Under full immersion, the strength loss rate increased progressively with exposure age. The loss rate was 10.04% at 30 days, rose to 11.20% at 60 days, and further increased to 22.25% at 90 days. This increasing trend reflects a continuous degradation of mechanical properties, which accelerated markedly in the later stage. Under semi-immersion with wetting–drying cycles, the strength loss rate was −8.06% at 30 days, indicating a strength gain. It then increased to 18.29% at 60 days and further to 29.03% at 90 days. The transition from negative to positive occurred between 30 and 60 days, after which the loss rate increased rapidly. The semi-immersion group showed a strength gain at 30 days, whereas the full-immersion group had already experienced a 10.04% loss at the same age. By 90 days, the strength loss rate of the semi-immersion group reached 29.03%, higher than the 22.25% loss of the full-immersion group, although statistical significance was not tested.

### 3.4. Linear Expansion

**Figure 5:**
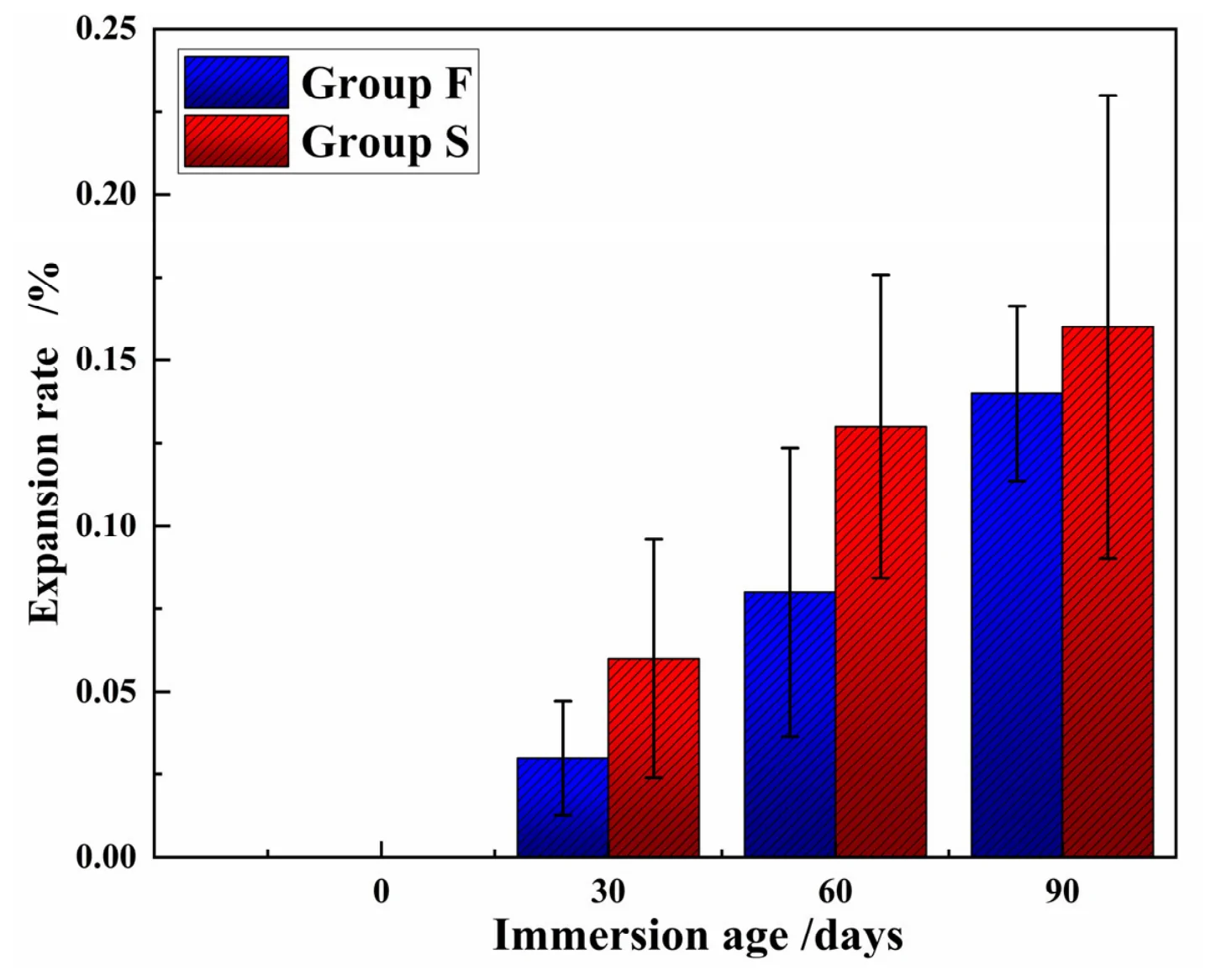
Linear expansion of LHPC paste under different exposure conditions.

Figure 5 shows the linear expansion of LHPC paste under the two exposure conditions. Under full immersion, the linear expansion rates at 30, 60, and 90 days were 0.03%, 0.08%, and 0.14%, respectively, showing a continuous increase with exposure age. Under semi-immersion with wetting–drying cycles, the corresponding values were 0.06%, 0.13%, and 0.16%, also exhibiting a consistent upward trend. In comparison, the semi-immersion group showed slightly higher expansion rates than the full-immersion group at all exposure ages, with differences ranging from 0.02 to 0.05 percentage points. Although both groups exhibited a clear increase in expansion over the 90-day period, the absolute values remained consistently low, with a maximum of only 0.16% at 90 days. This indicates that despite the ongoing chemical attack, the low C_3_A content of LHPC is expected to limit ettringite-related expansion, keeping the expansion at a minimal level throughout the exposure period [32].

### 3.5. Microstructural and Elemental Analysis

**Figure 6:**
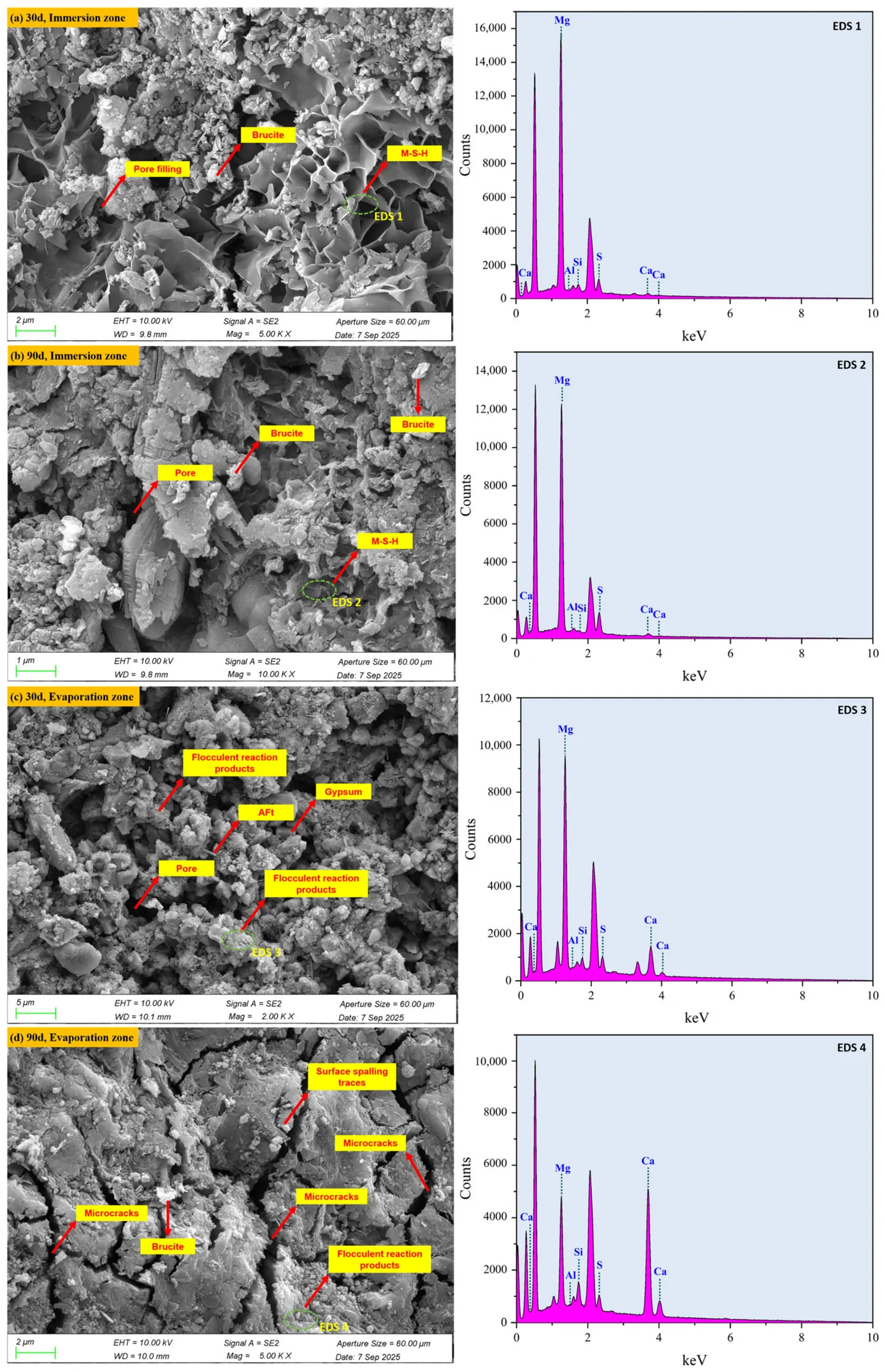
SEM images and corresponding EDS spectra of semi-immersed specimens in immersion and evaporation zones.

Based on the macroscopic results, the semi-immersed specimens exhibited more severe deterioration than the fully immersed ones and were therefore selected for microstructural observation. Figure 6 presents SEM images of the semi-immersed specimens in the immersion and evaporation zones after 30 and 90 days of exposure.

At 30 days, the immersion zone exhibited abundant tabular gypsum crystals intergrown with granular brucite, while no needle-like ettringite crystals were observed. The evaporation zone was covered with flocculent reaction products and showed a relatively dense structure. In both zones, the attack products mainly filled the pores, and no obvious microcracks were observed. At 90 days, the number of gypsum crystals in the immersion zone increased markedly, with some areas showing clustered growth, and the overall structure became looser. In the evaporation zone, the combined effects of chemical attack and salt crystallization led to microcracks and surface spalling traces, with greater loss of compactness than that observed in the immersion zone. No typical needle-like ettringite crystals were observed in either zone. Based on the SEM observations, secondary products continued to form and accumulate with increasing exposure age, while the compactness of the matrix progressively declined. The deterioration in the evaporation zone was more pronounced than that in the immersion zone, as evidenced by the more extensive microcracks and surface spalling traces. The EDS spectra were collected from the typical reaction product regions in the immersion and evaporation zones, including the flocculent products and M-S-H-rich areas. These spectra show a clear enrichment of Mg and Si and a depletion of Ca in the evaporation zone, which is consistent with the conversion of C-S-H to M-S-H. In the immersion zone, this trend is less pronounced. Elemental mapping could not be performed due to sample limitations; therefore, the EDS results are presented as point/area spectra, and the collection positions are marked in Figure 6.

### 3.6. Phase Composition

**Figure 7:**
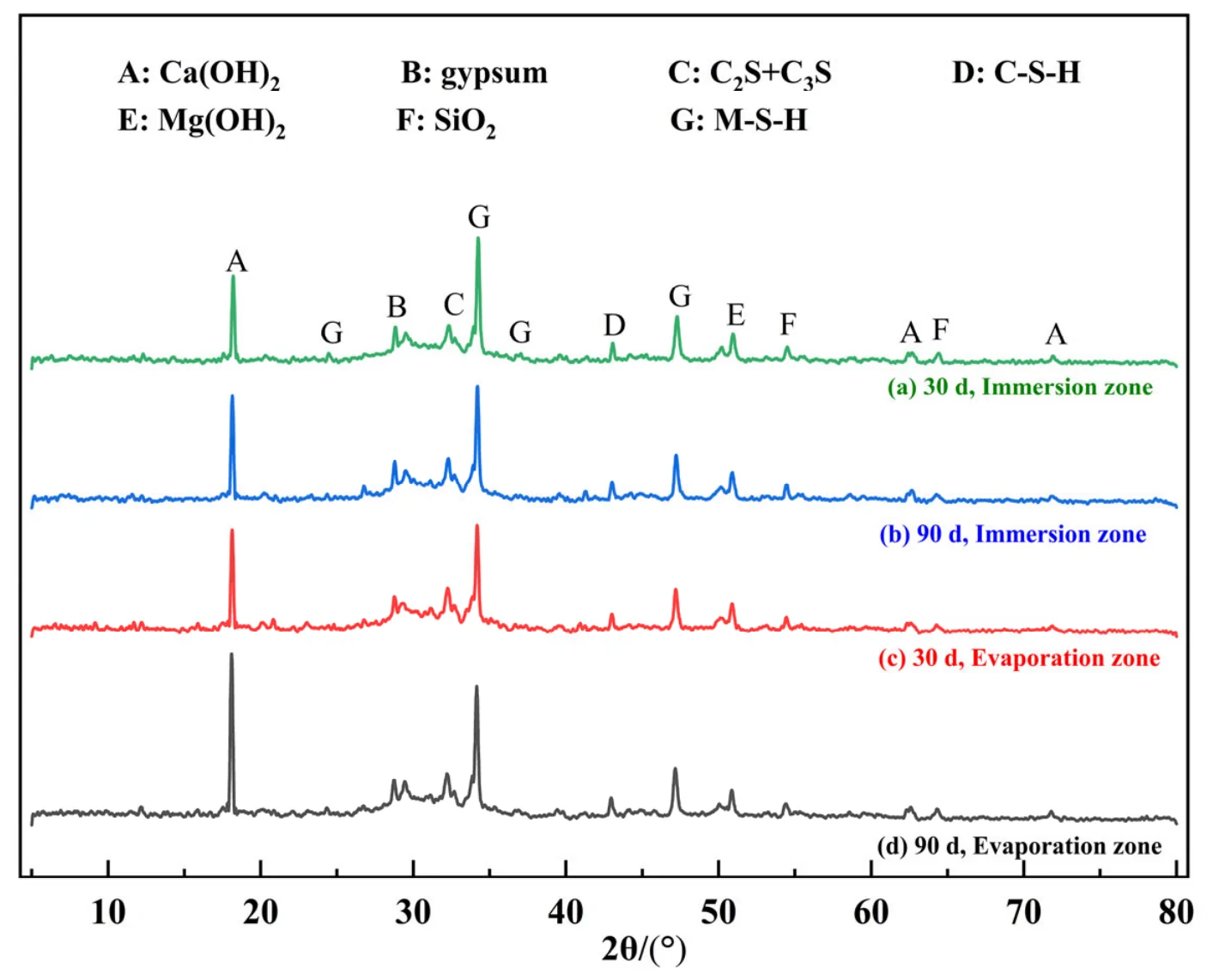
XRD patterns of semi-immersed specimens in immersion and evaporation zones.

Figure 7 shows the XRD patterns of the semi-immersed specimens in the immersion and evaporation zones. At 30 days, both zones exhibited a broad diffuse hump in the low-angle region, which is consistent with the presence of poorly crystalline M-S-H, along with distinct diffraction peaks of Mg(OH)_2_ and gypsum. No obvious diffraction peaks of ettringite were detected. The peaks of Ca(OH)_2_ were present in both zones. At 90 days, the broad diffuse hump remained visible in both zones; however, its relative intensity was not quantitatively compared because M-S-H is poorly crystalline and may overlap with C-S-H and other amorphous phases. The Ca(OH)_2_ peaks remained visible in both zones, with no significant reduction in intensity compared with the 30-day patterns. The gypsum peaks showed increased intensity relative to those at 30 days. Throughout the exposure period, weak ettringite peaks cannot be excluded. Because M-S-H is poorly crystalline and its diffuse scattering may overlap with C-S-H and other amorphous phases, the XRD patterns alone cannot be used to quantify the relative amount of M-S-H between zones or exposure ages.

### 3.7. Pore Structure

**Figure 8:**
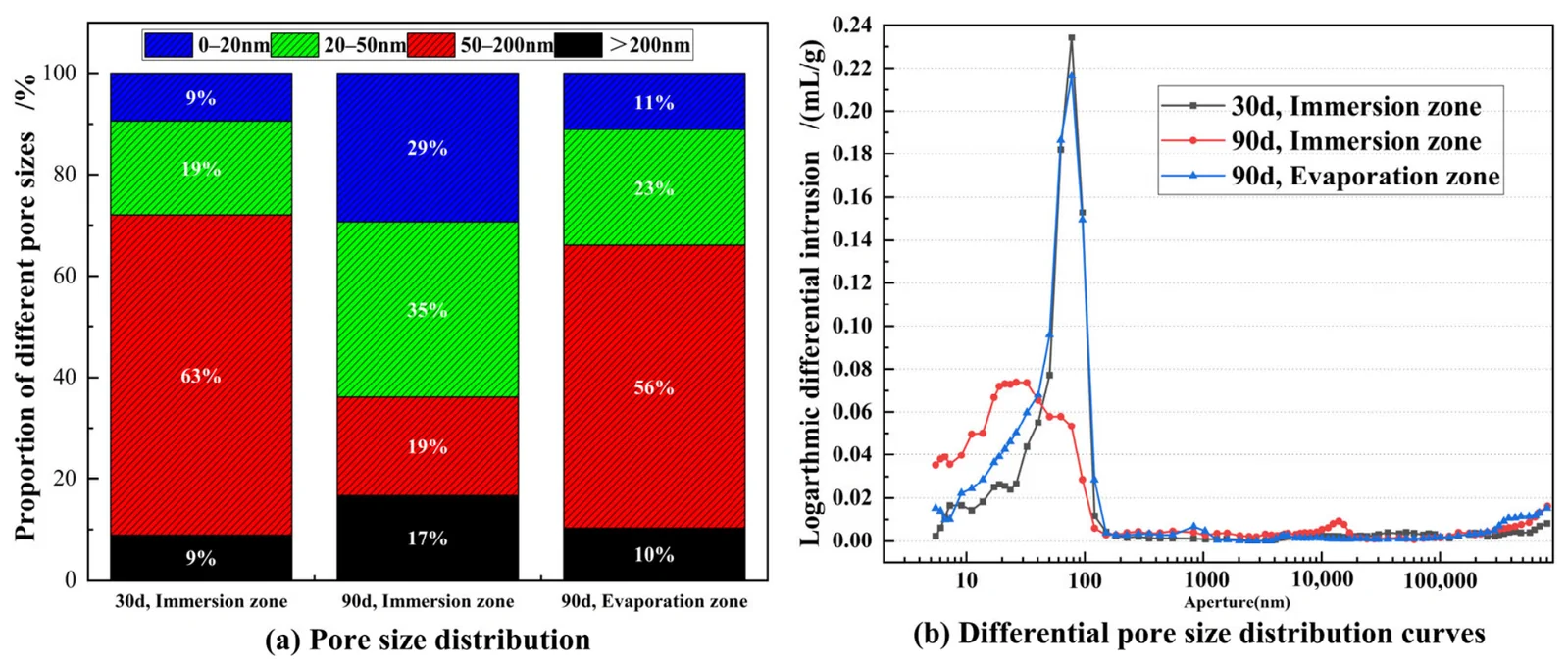
Pore size distribution of semi-immersed specimens in immersion and evaporation zones.

Figure 8 shows the pore size distribution of the semi-immersed specimens in the immersion and evaporation zones. At 30 days, the pores in the immersion zone were predominantly mesopores ranging from 50 to 200 nm, accounting for 63% of the total porosity, while gel pores below 20 nm constituted only 9%. The derivative curve exhibited a sharp unimodal shape with its peak in the mesopore range, indicating preferential filling of smaller gel pores by early-stage reaction products. At 90 days, the pore structure of the immersion zone underwent a substantial redistribution. The proportion of 50–200 nm mesopores decreased from 63% to 19%, while the <20 nm fraction increased from 9% to 29%, the 20–50 nm fraction increased from 19% to 35%, and the >200 nm fraction increased from 9% to 17%. The derivative curve changed from a sharp unimodal peak at 30 days to a broad multimodal form. This evolution suggests that repeated dissolution and recrystallization of attack products redistributed the pore system, rather than uniformly coarsening it. It should be noted that this redistribution does not contradict the overall open porosity reduction reported in Section 3.2, as the two metrics reflect different aspects of pore structure.

Due to the limited sample volume obtainable from the thin evaporation zone at 30 days, the pore-size distribution for this region is presented only at 90 days. At 90 days, the evaporation zone contained 56% mesopores of 50–200 nm and only 10% macropores larger than 200 nm, compared with 17% macropores in the immersion zone. This spatial comparison suggests that macropore development is not directly proportional to deterioration severity. However, because the pore structure was characterized on surface-layer samples while compressive strength was measured on the whole specimen, the contribution of pore-structure changes to the overall strength loss cannot be quantitatively isolated in this study. The open porosity reported in Section 3.2 reflects the open pore volume of the whole specimen, whereas the MIP results describe the pore-size distribution of the surface layer. The decrease in open porosity is mainly attributed to pore filling by reaction products, while the redistribution of pore sizes is attributed to dissolution and recrystallization. These two metrics characterize different aspects of the pore system and should not be treated as directly interchangeable. The decrease in open porosity should not be interpreted as an absence of deterioration. It mainly reflects pore filling by reaction products within the whole specimen, while the overall deterioration is evidenced by strength loss, microcracking, and spalling.

## 4. Discussion

**Figure 9:**
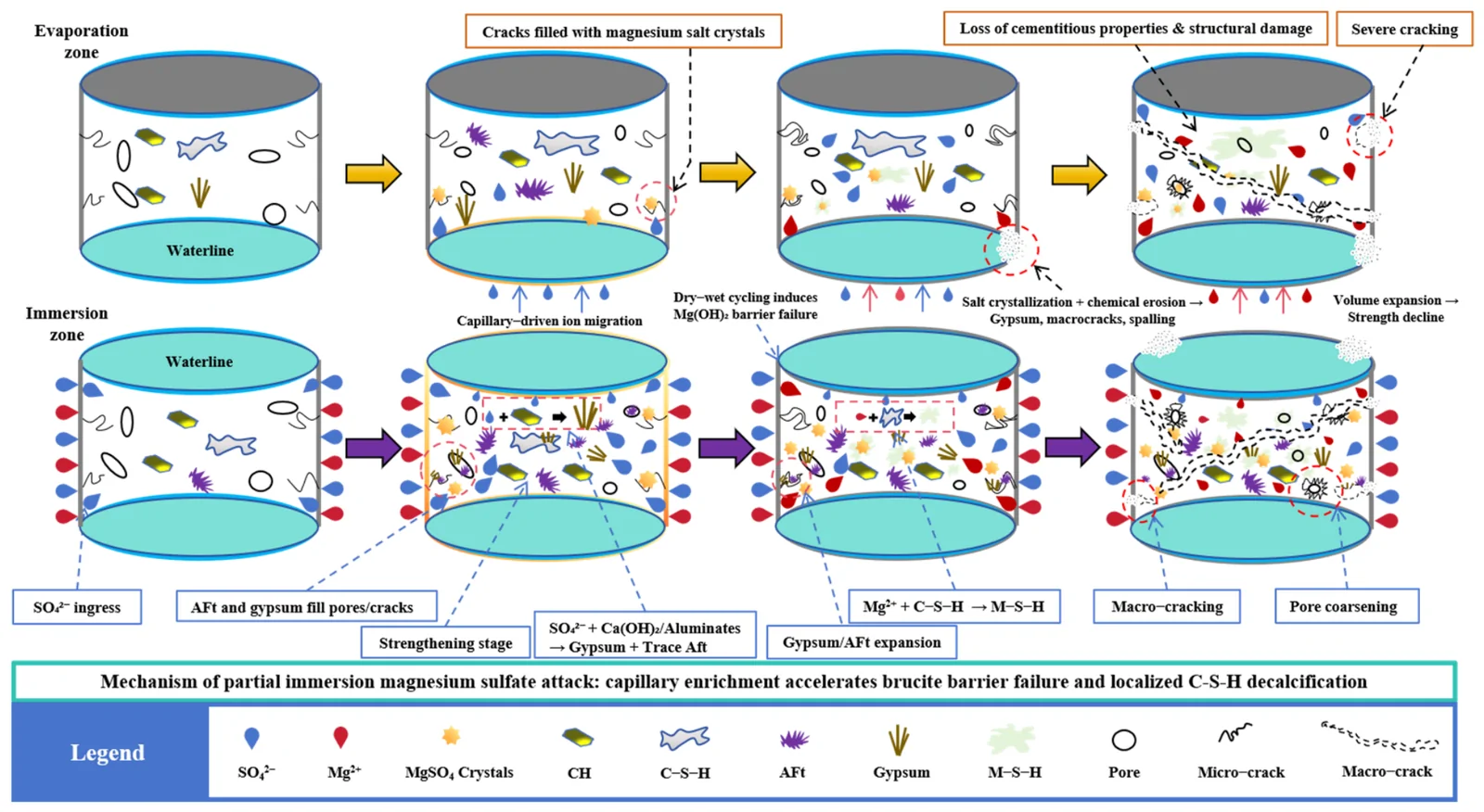
Schematic of deterioration mechanism under semi-immersion with wetting–drying cycles in MgSO_4_ solution.

**Figure 10:**
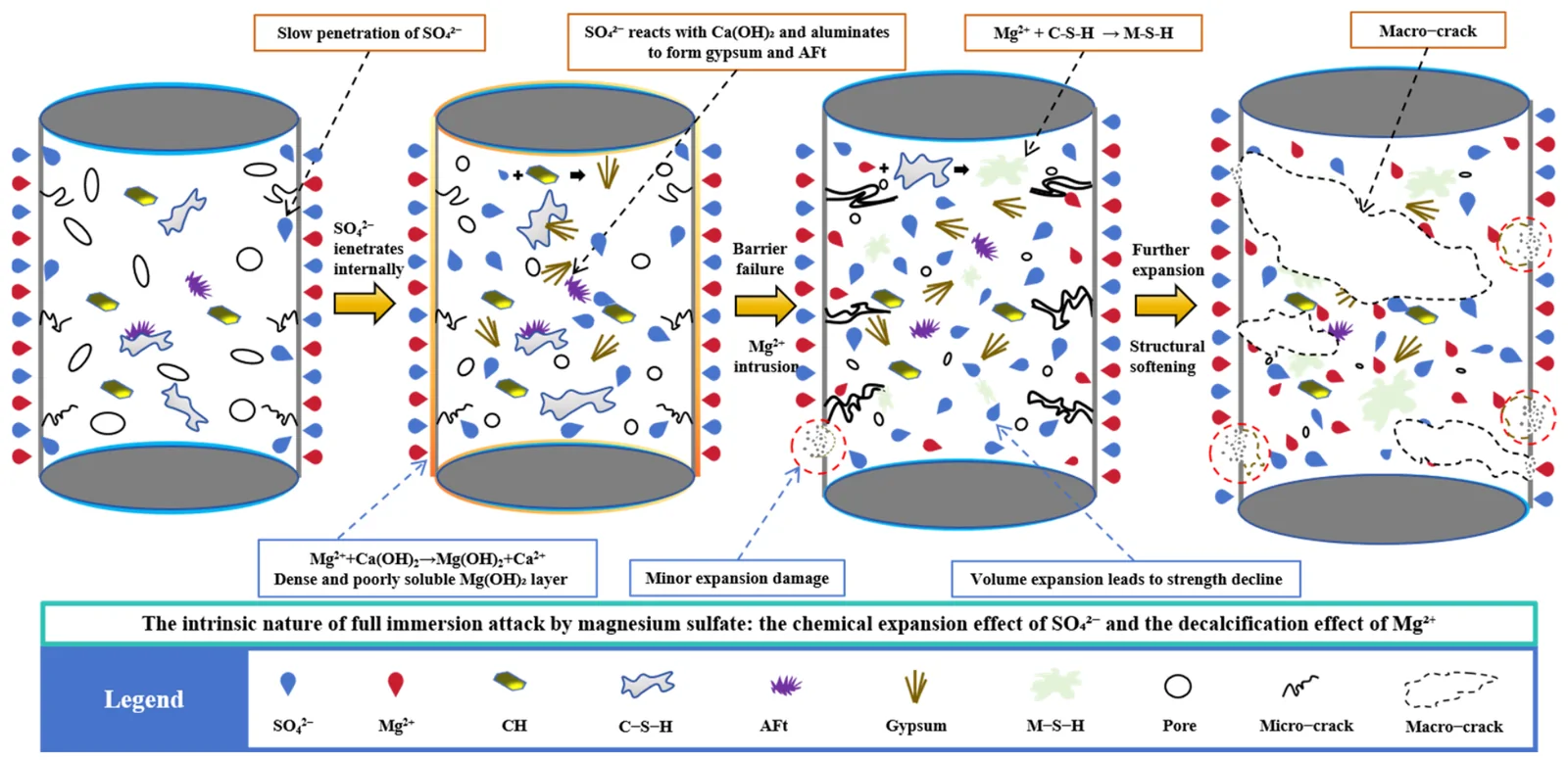
Schematic of deterioration mechanism under full immersion in MgSO_4_ solution.

The deterioration mechanism of cement-based materials subjected to magnesium sulfate attack is fundamentally different from that of sodium sulfate attack. Sodium sulfate attack is characterized by expansion and cracking induced by the crystallization of expansive products, namely ettringite and gypsum. In contrast, the primary threat of magnesium sulfate attack is the direct assault of Mg^2+^ on C-S-H gel, which is converted into non-cementitious M-S-H via Ca^2+^ exchange, ultimately leading to loss of cementitiousness and matrix softening [33]. Figure 9 schematically illustrates the deterioration mechanism under semi-immersion with wetting–drying cycles, based on the microscopic characterizations in this work. Figure 10 presents the corresponding mechanism under full immersion, which is redrawn based on data from our previous studies [2,9], serving as a background reference for comparison. Notably, the proposed degradation mechanism is an interpretation based on the macroscopic and microscopic observations in this study, combined with the previous literature. Direct measurements of ion transport, brucite barrier thickness, Mg^2+^ concentration profiles, ion penetration depth, and concentration enrichment along the specimen height were not performed. Thus, the proposed barrier formation and failure sequence is best viewed as a plausible interpretation, not a direct experimental demonstration. The proposed transport mechanism is therefore an interpretation based on the spatial distribution of degradation products and microstructural observations.

Under semi-immersion with wetting–drying cycles, the deterioration process exhibits a clear two-stage feature, with early strengthening followed by later softening. In the early stage, Mg^2+^ is inferred to react preferentially with Ca(OH)_2_ to form a dense brucite layer on the paste surface and pore walls. This layer is believed to act as an ion transport barrier, potentially impeding further ingress of SO_4_^2−^ and Mg^2+^ [34,35]. Concurrently, SO_4_^2−^ combines with Ca^2+^ to form gypsum. The brucite, gypsum, and residual C-S-H synergistically fill the pores, refining the pore structure. This strengthening stage is macroscopically manifested by a reduction in porosity to 4.73% at 30 days, an increase in compressive strength, and a mass loss rate of 1.32%. Both XRD and SEM confirmed the presence of brucite and gypsum at 30 days, while MIP results indicated that the degradation products preferentially filled the gel pores, concentrating the pore size distribution in the mesopore range. However, the brucite barrier is inferred to be gradually compromised under the combined action of ongoing chemical attack and cyclic wetting–drying stresses. Once the barrier is compromised, Mg^2+^ is believed to penetrate the matrix more readily, triggering decalcification of C-S-H and its conversion into non-cementitious M-S-H. This agrees with the observed increase in M-S-H-related features and the strength loss at later ages, but the transport process itself was not directly monitored [36,37]. This transformation is evidenced by the broad diffuse hump in the low-angle region of the XRD patterns, together with the SEM-EDS results presented in Figure 6, which are consistent with the formation of M-S-H, as well as the loosening of the matrix structure observed in SEM images. The XRD patterns show that CH remained present at 90 days, indicating that CH was not completely consumed. Correspondingly, the strength loss rate rose sharply to 29.03% at 90 days, and the mass loss rate reached 3.72%. MIP data further showed that macropores (>200 nm) in the immersion zone increased from 9% to 17%, whereas pore structure coarsening in the evaporation zone was far less pronounced. This contrast suggests that chemical degradation of cementitious phases is likely an important contributor to the strength loss of the semi-immersed specimens, rather than physical pore coarsening alone. However, because the microstructural analyses were performed on surface-layer samples while compressive strength was measured on the whole specimen, the contribution of each zone to the overall strength loss cannot be quantitatively isolated in this study [38]. Overall, these results suggest that under semi-immersion with wetting–drying cycles, chemical degradation and physical damage occur simultaneously rather than sequentially, and that the former is likely a major contributor to strength loss. This mechanism, however, is contingent upon the transport mode of aggressive ions, which differs fundamentally between the two exposure conditions. Also, under semi-immersion and wetting–drying cycles, chemical sulfate attack and physical salt crystallization occur at the same time. The relative contribution of each process may vary with depth and exposure time, and cannot be fully separated in this study.

As a reference, the full immersion condition undergoes the same fundamental deterioration process as the semi-immersion condition, with both experiencing the formation and subsequent failure of the brucite barrier, eventually losing their cementitiousness owing to M-S-H formation [39]. However, significant differences exist in the temporal progression and severity of deterioration, with the full immersion condition serving as a baseline for understanding the accelerated deterioration under semi-immersion. Under full immersion, ions penetrate the matrix through steady-state diffusion driven by concentration gradients. Under full immersion, a brucite barrier forms steadily. However, the ion supply becomes diffusion-limited, and the reaction products may hinder further transport. Consequently, only limited densification is achieved and no early strength gain occurs; the strength loss rate reached 10.04% at 30 days. After the gradual failure of the barrier, it rose to 22.25% at 90 days, and the mass loss rate was 4.41%. In contrast, under semi-immersion, capillary suction coupled with evaporative concentration is considered the dominant transport mechanism. The wetting–drying cycles are inferred to continuously supply Mg^2+^ through capillary action. SEM observations suggest a dense surface layer in the semi-immersed specimens at 30 days. However, this strengthening effect has a double-edged nature. While the barrier may be more effective initially, the alternating wetting–drying stresses and repeated gypsum crystallization may cause it to fail earlier than under full immersion. This is inferred from the different deterioration trends between the two exposure conditions rather than from a direct microscopic comparison, because the microstructural characterization was primarily performed on semi-immersed specimens. Once the barrier is compromised, Mg^2+^ is believed to penetrate more readily, and deterioration intensifies rapidly, ultimately resulting in more severe damage than that under full immersion [40]. This process is further supported by the broad diffuse hump in the low-angle region and the SEM-EDS results, which are consistent with M-S-H formation in the evaporation zone at 90 days.

The mineral composition of LHPC exhibits a clear double-edged effect in magnesium-rich environments. The low C_3_A content of LHPC is expected to limit ettringite formation, although the presence of small amounts of poorly crystalline ettringite cannot be completely ruled out by XRD. This keeps expansion consistently low throughout the exposure period and avoids the expansion-induced cracking typical of sodium sulfate attack, while the abundant C-S-H gel derived from hydration of the high C_2_S content provides a substantial substrate for Mg^2+^-induced decalcification, making structural softening the main deterioration mode [41]. Under semi-immersion with wetting–drying cycles, the capillary suction and evaporative concentration mechanism amplifies Mg^2+^ transport efficiency, rendering the softening edge of the double-edged sword even sharper [42,43]. Building on these findings, the resistance of low-heat cement concrete in magnesium-rich environments to decalcification can be enhanced by incorporating supplementary cementitious materials to lower the Ca/Si ratio of C-S-H, or by applying surface protection to block the ingress of Mg^2+^. Furthermore, although expansion rate is widely adopted as an evaluation index for sulfate attack in current standards, the present results indicate that this index is of limited applicability to magnesium sulfate attack. The chemical degradation of cementitious phases in the evaporation zone is likely an important contributor to the overall strength loss of the semi-immersed specimens. Therefore, strength degradation is recommended as a performance indicator, while M-S-H formation should be regarded as a mechanistic parameter requiring further quantitative validation.

While the accelerated regime used in this study is intended for mechanistic investigation rather than direct prediction of field service life, the coupled effects of MgSO_4_ attack and 80 °C drying could not be fully separated in this study, and the microstructural sampling was limited to the immersion and evaporation zones. Future work will therefore include a control group exposed to wetting–drying cycles without MgSO_4_ and more systematic sampling along the specimen height, from the immersion zone through the transition zone to the evaporation zone, to further substantiate the deterioration gradient. It should also be noted that statistical significance testing was not performed, and the conclusions should therefore be regarded as indicative rather than statistically validated.

## 5. Conclusions

This study investigated the deterioration behavior and mechanisms of LHPC paste under MgSO_4_ attack, with a focus on the semi-immersion regime coupled with wetting–drying cycles, using full immersion as a macroscopic reference. The main conclusions are as follows:

- MgSO_4_ attack on LHPC paste showed a two-stage feature of early strengthening followed by later softening. The early formation of a brucite layer is inferred to refine the pore structure, while the later transformation of C-S-H into non-cementitious M-S-H is inferred to contribute to matrix softening. M-S-H formation is a key feature distinguishing magnesium sulfate attack from sodium sulfate attack.
- The exposure condition substantially governs the deterioration process. Under full immersion, the strength loss reached 22.25% at 90 days with no strengthening stage. Under semi-immersion with wetting–drying cycles, an early strength gain occurred, consistent with brucite layer formation. The later strength loss, reaching 29.03% at 90 days, is inferred to result from the compromise of this layer and the subsequent conversion of C-S-H to M-S-H.
- The mineral composition of LHPC exhibits a double-edged effect in magnesium-rich environments. The low C_3_A is expected to limit ettringite-induced expansion, although poorly crystalline ettringite cannot be completely ruled out by XRD. The abundant C-S-H derived from high C_2_S hydration is expected to provide a substantial substrate for Mg^2+^-induced decalcification, making structural softening a major deterioration mode.
- The predominant deterioration mechanism is considered to be the loss of cementitiousness caused by M-S-H formation, rather than pore coarsening alone. This interpretation requires further validation because the microstructural analyses were performed on surface-layer samples, and the contribution of the evaporation zone to the overall strength loss cannot be quantitatively isolated.
- For the LHPC paste and exposure conditions tested here, strength degradation is recommended as a performance indicator while M-S-H formation should be regarded as a mechanistic parameter requiring further quantitative validation. Statistical significance testing was not performed, and the conclusions should therefore be regarded as indicative rather than statistically validated.

## Figures and Tables

**Table 1:** Chemical and mineral composition of cement (%).

CaO	SiO_2_	Al_2_O_3_	Fe_2_O_3_	MgO	SO_3_	R_2_O *	C_3_S	C_2_S	C_3_A	C_4_AF
58.74	22.82	3.55	4.28	4.99	2.43	0.39	28.7	43.9	2.1	13.0

* R_2_O = Na_2_O + 0.658K_2_O.

**Table 2:** Physical and mechanical properties of the cement.

Density/(g·cm^−3^)	Specific Surface Area/(m^2^·kg^−1^)	Setting Time/Min		Soundness	Compressive Strength/MPa		
		Initial	Final		7 d	28 d	90 d
3.21	332	233	343	Conforming	25.4	53.6	69.8

**Table 3:** Exposure conditions for the two test groups.

Group	Exposure Condition	Cycle Parameters
F	Full immersion	None
S	Semi-immersion at half height	15.5 h immersion, 0.5 h air drying, 6 h oven drying at 80 °C ± 5 °C, and ~2 h cooling and transfer

## Data Availability

The original contributions presented in this study are included in the article. Further inquiries can be directed to the corresponding author.
